# Effect of Different Treatments on Retinal Thickness Changes in Patients With Multiple Sclerosis: A Review

**DOI:** 10.1111/cns.70225

**Published:** 2025-01-24

**Authors:** Armin Adibi, Iman Adibi, Milad Javidan

**Affiliations:** ^1^ Department of Neurology Isfahan University of Medical Sciences Isfahan Iran; ^2^ Neuroscience Research Center Isfahan University of Medical Sciences Isfahan Iran

**Keywords:** drug therapy, multiple sclerosis, optic coherence tomography, retina

## Abstract

**Background:**

Multiple sclerosis (MS) is an autoimmune disorder affecting the central nervous system, with varying clinical manifestations such as optic neuritis, sensory disturbances, and brainstem syndromes. Disease progression is monitored through methods like MRI scans, disability scales, and optical coherence tomography (OCT), which can detect retinal thinning, even in the absence of optic neuritis. MS progression involves neurodegeneration, particularly trans‐synaptic degeneration, which extends beyond the initial injury site. This review focuses on the impact of different MS treatments on retinal thickness as assessed by OCT.

**Results:**

Injectable drugs, such as interferon beta and glatiramer acetate (GA), have a relatively modest impact on retinal atrophy. Oral medications like Fingolimod, Teriflunomide, and Dimethyl fumarate also have different impacts on retinal thickness. Fingolimod has been shown to protect against retinal thinning but may lead to macular edema. DMF‐treated patients had less ganglion cell–inner plexiform layer thinning than GA‐treated patients but more thinning compared to natalizumab‐treated patients and healthy controls. Teriflunomide's impact on retinal layers remains unexplored in human studies. Monoclonal antibodies, including Alemtuzumab, Rituximab, Ocrelizumab, and Natalizumab, had protective effects on retinal layer atrophy. Alemtuzumab‐treated patients showed significantly less atrophy compared to interferon‐ and GA‐treated patients. Rituximab initially increased atrophy rates in the first months but subsequently demonstrated potential neuroprotective effects. Ocrelizumab slowed the rate of inner nuclear layer thinning in progressive forms of the disease. Natalizumab is considered the most effective in reducing retinal layer atrophy, particularly the peripapillary retinal nerve fiber layer.

**Conclusions:**

It's important to note that the effectiveness of these treatments may vary depending on MS subtype and individual factors. Future research should explore the long‐term effects of these treatments on retinal layers and their correlations with overall disease progression and disability in MS patients.

## Graphical abstract

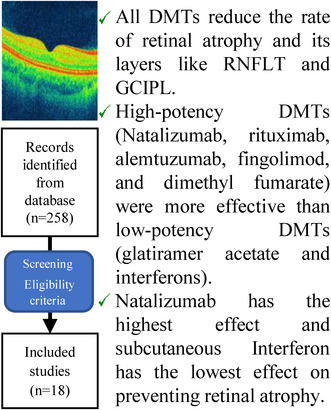

## 1 Introduction

Multiple sclerosis (MS) is an autoimmune disorder of the central nervous system and a significant cause of non‐traumatic disability, among young adults. This disorder is characterized by distinctive features such as demyelination, gliosis, and the formation of lesions dispersed throughout the central nervous system, often prominently visible on magnetic resonance imaging (MRI) [1, 2, 3, 4].

In the course of the disease, MS pathophysiologic characteristics change from acute inflammatory phase to chronic degenerative state. During this course, lesions can enter a phase of remyelination, resolution of inflammation without repair, or the persistence of a “smoldering” state, characterized by concurrent inflammation and myelin degeneration [5]. Neurodegenerative changes play a central role in the development of irreversible clinical disability, significantly affecting patients' walking ability, cognition, fatigue, and vision [6].

Radiographic measures of disease progression involve the development and enlargement of T2 lesions [7]. Complementing these MRI‐based measures, optical coherence tomography (OCT) offers insights into changes in the anterior visual system, influenced by factors such as acute optic neuritis (AON) attacks and retrograde axonal atrophy [8]. OCT serves as an invaluable tool for the monitoring of MS patients, detecting retinal nerve fiber layer (RNFL) thinning even in the absence of optic neuritis (ON) and assessing treatment response [9]. This imaging technique provides a rapid, non‐invasive, cost‐effective, well‐tolerated, and reliable means of quantifying distinct retinal layers [10, 11, 12, 13]. Optic nerve lesions were incorporated in revised McDonald criteria, which increased its specificity and accuracy [14]. Factors influencing RNFL thinning include age, disease subtype, prior ON history, the use of high‐potency disease‐modifying treatments, and their interactions [15, 16]. Additionally, accelerated thinning of the ganglion cell–inner plexiform layer (GCIPL) and peripapillary retinal nerve fiber layer (pRNFL) is associated with the progression of clinically isolated syndrome (CIS) to MS [17].

The mechanism for retinal atrophy is believed to result from retrograde trans‐synaptic degeneration (TSD), which adds complexity to the understanding of MS pathogenesis [18]. In TSD, injury to a neuron or axon leads to the degeneration of synaptically connected axons and neurons, either in an anterograde or retrograde direction. This mechanism extends beyond the initial injury site, impacting the central nervous system far from its origin [19, 20, 21, 22].

TSD can cause structural changes in the anterior visual pathway, as evidenced by retinal OCT, with thinning observed in peripapillary retinal nerve fiber layer (pRNFL), macular ganglion cell–inner plexiform layer (GCIPL), and inner plexiform retinal layer (IRL). Notably, these layers exhibit more pronounced thinning in progressive forms of MS, in contrast to the relapsing–remitting subtype. Conventional anti‐inflammatory disease‐modifying treatments appear to have limited efficacy in the progressive forms [23, 24, 25].

Injury to the posterior visual pathway, along with trans‐synaptic degeneration, subclinical optic neuritis, and disruption of the blood–brain barrier by active lesions—resulting in the extension of inflammation into the visual pathways—is thought to contribute to neuroaxonal loss in the retina [26, 27, 28].

Additionally, MS is commonly accompanied by ON. In cases of AON, unmyelinated pRNFL thickness increases due to acute inflammation within the optic nerve, accompanied by detectable inner nuclear layer (INL) thickness increase. However, with the resolution of inflammation, retrograde degeneration sets in, resulting in axonal loss in pRNFL and the loss of retinal ganglion cell bodies in the GCIPL, as evidenced by rapid pRNFL and GCIPL thinning on OCT [29, 30, 31]. Concomitantly, INL and outer nuclear layer (ONL) thinning occurs as GCIPL thickness decreases [29, 30]. Each episode of ON in MS patients is associated with a more significant reduction in GCIPL thickness, and over the long term, GCIPL thickness may be lower in ON eyes compared to non‐ON eyes [32]. The dynamic changes in retinal layer thickness observed after AON appear to be confined to the first 4–6 months, during which the pRNFL and GCIPL layers undergo rapid thinning. This suggests a cascade of neuroaxonal degeneration triggered by AON, potentially extending to the optic tracts and culminating in the loss of third‐order neurons that project from the lateral geniculate nucleus of the thalamus to the primary visual cortex in the occipital lobe [33, 34]. Furthermore, bipolar cells, which constitute the majority of INL cells, may also be implicated in TSD [35]. In addition to axonal loss in the pRNFL and the loss of retinal ganglion cell bodies in the GCIPL caused by TSD, other mechanisms—such as oxidative stress, mitochondrial dysfunction, and neurodegeneration—also contribute to retinal layer atrophy [36, 37].

INL and ONL thinning are associated with white matter lesions and are accelerated in progressive forms of MS. Unlike GCIPL and pRNFL, these changes are not influenced by treatments [25] and have been linked to increased MS disability [38, 39, 40]. ONL thinning is associated with faster whole‐brain and substructure atrophy, distinct from the faster GCIPL thinning and suggestive of global neurodegeneration in the MS brain [22].

GCIPL thinning is associated with thalamic atrophy, implying a larger magnitude of retinal tissue loss after AON and its link to anterograde trans‐synaptic neuroaxonal degeneration [22]. Thalamic atrophy has emerged as a crucial biomarker of neurodegeneration in MRI, associated with clinical outcomes and extraocular complications affecting sensory, motor, and cognitive functions [41, 42, 43]. GCIPL thickness has been shown to be associated with total disability and decision‐making in MS patients, as well as brain atrophy and visual function. Also, GCIPL atrophy is linked to intrathecal immune activation and functions as an independent risk factor for increased disability [44].

Retinal layer atrophy is most prominent in the early stages of the disease, coinciding with the highest rate of neuroaxonal loss [28, 45].

Notably, retinal layers such as the pRNFL and GCIPL are closely linked to various disease features, including disease duration, brain atrophy, neurological and visual function, global CNS degeneration, clinical disability, and cognitive performance. Furthermore, the thickness of these layers can predict future relapses, disease activity, disability progression, inflammation, and visual impairment [46, 47, 48, 49], Therefore, retinal thickness and its layers, as assessed by OCT, are valuable surrogate biomarkers for neurodegeneration, neuroaxonal damage, and disease activity [49].

Since, thicknesses of certain retinal layers of retina are decreased during progress of MS which can be a window to the brain and this thinning is discoverable by OCT, which can serves as a valuable biomarker for assessing the effects of MS drugs [23, 50], this review article aims to provide an up‐to‐date overview of the current evidence regarding the impact of existing treatments on retinal thickness in MS patients, emphasizing the critical role of OCT in monitoring disease progression and treatment response.

## 2 Methods

This review aimed to gather all the studies that have longitudinally assessed retinal thickness and volume in MS patients who were undergoing disease‐modifying therapy (DMT) treatment. To conduct the literature review, we performed electronic searches using MEDLINE, Embase, and Web of Science Core Collection databases utilizing the following keywords in all fields: (“retina” OR “macula” OR “optic coherence tomography”) AND (“fingolimod” OR “ozanimod” OR “siponimod” OR “ponesimod” OR “laquinimod” OR “cladribine” OR “teriflunomide” OR “dimethyl fumarate” OR “diroximel fumarate” OR “monomethyl fumarate” OR “interferon” OR “glatiramer acetate” OR “ofatumumab” OR “natalizumab” OR “rituximab” OR “ocrelizumab” OR “alemtuzumab” OR “mitoxantrone”) AND (“multiple sclerosis”), starting from their inception until 1st September 2022. After eliminating duplicate studies, two independent screeners reviewed the references and extracted data related to DMTs and the reported outcome measures in each study. Afterward, the titles and abstracts of studies were reviewed, and potentially relevant full‐text studies were selected. The following inclusion criteria were applied to decide if papers would be included: The study used a longitudinal design; the study measured the retinal layer or one of its layers (includes GCIPL or pRNFL, or INL or ONL), and the research involved humans. The exclusion criteria for studies were as follows: papers that did not focus on retinal thickness, animal studies, papers unrelated to DMTs, reviews, case reports, book chapters, and editorials, and those that did not provide sufficient data on retinal thickness. Additionally, the screeners manually searched the references in these articles to ensure inclusivity.

Any discrepancy between two reviewers was addressed by negotiation with the third reviewer.

The Joanna Briggs Institute's (JBI's) critical appraisal tool was employed to assess the quality of the studies. Based on the checklist questions, each article was assigned a rating of “yes,” “no,” “unclear,” or “not applicable” [51]. A score of 1 was given for each criterion that was clearly met, while 0 was assigned for unmet criteria, with a total possible score of 8. Studies were then categorized according to their scores: those scoring 7–9 were deemed high quality, 4–6 were considered moderate quality, and 0–3 were classified as low quality.

## 3 Results

**FIGURE 1:**
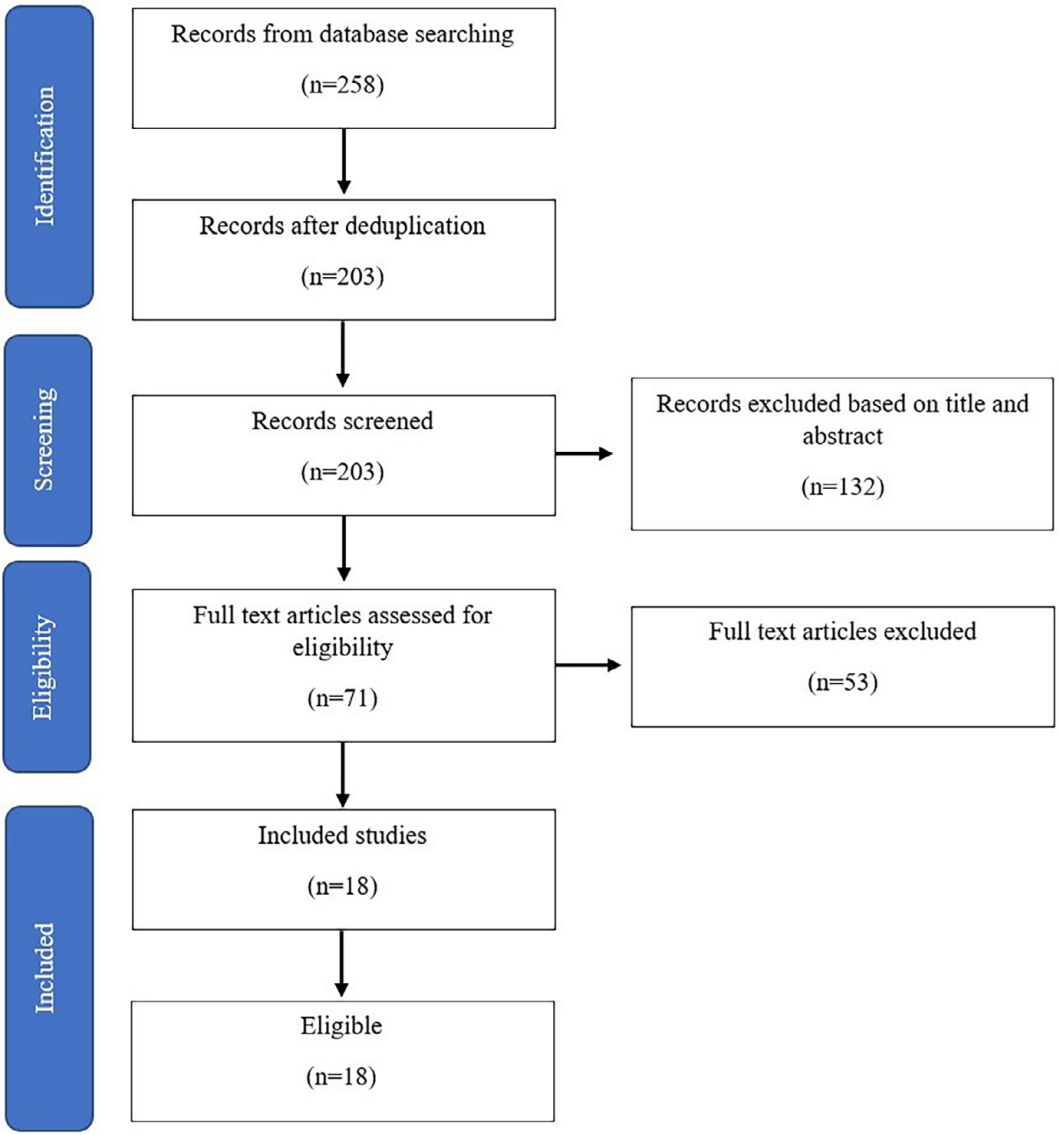
PRISMA flowchart for study identification and selection.

Studies that did not fulfill the eligibility criteria (not focusing on retinal thickness or macular volume, animal studies, not related to oral DMTs, reviews, case reports, book chapters, editorials) and those that did not provide sufficient data on retinal thickness were excluded. As a result, 18 studies were included in this review (Figure 1). These studies assessed retinal thickness in MS patients undergoing DMT treatment. The majority of these studies (*n* = 8) provided data on S1P modulators fingolimod (FTY), glatiramer acetate (*n* = 8), and interferons (*n* = 7). Additionally, we found seven studies that reported data on other drugs. Characteristics of included articles are demonstrated in Table 1. Table 2 provided an overview comparing the effectiveness of high‐potency DMTs against low‐ and medium‐potency DMTs and healthy controls in terms of retinal thickness. The focus was primarily on pRNFL and GCIPL measurements, as these were more frequently utilized and showed stronger correlation with disease specificity.

**TABLE 1:** Characteristics of included studies.

Author (year)	Study design	Sample size	Course (Disease phenotype)	Groups	Route and dosing (duration)	Outcome	Assessment	Main finding	Other findings	Risk of bias assessment
El Ayoubi et al. (2021)	Prospective observational study	128	RRMS	FTY vs. IFN vs. Healthy	24 months	Rate of GCIPL, pRNFL, and macular volume changes in RRMS followed up on FTY or IFN	OCT	Lower atrophy rate than IFN with FTY and a similar rate with Healthy		High
Jakimovski et al. (2021)	Longitudinal cohort study	144	109RRMS 35PMS	Medium‐potent DMTs vs. high‐potent DMTs	60 months	Rate of pRNFL changes in MS	OCT	Greater thinning of pRNFL in medium‐potent DMTs (IFN, GA, DMF, TFD) compared to high‐potent DMTs (NTZ, FTY, ATZ, CLA OCR)	Age, disease subtype, history of prior MSON, use of DMTs effects on retinal layer	Moderate
You et al. (2021)	Cohort study	130	105RRMS 25 healthy	IFN β vs. GA vs. FTY vs. NTZ	48 months	Effect of Different DMTs on RNFL and GCIPL Thinning in MS	OCT	Highest atrophy rate in IFN compared with other DMTs	Lower rates of RNFL and GCIPL thinning in high‐potency DMTs (including FTY, DMF, NTZ, ATZ, RTX, and Ocrelizumab compared with platform therapies (IFN‐β and GA)	High
Button et al. (2017)	Retrospective cohort	402	RRMS	IFNSC vs. IFNIM vs. NTZ vs. GA	More than 1 year	Effects of different DMTs on GCIPL, pRNFL, INL, ONL, and AMT in RRMS	OCT	IFNSC displayed the highest rate of GCIPL atrophy compared to other DMTs	Natalizumab reduced the GCIPL atrophy rate more than all other DMTs	High
Chan et al. (2019)	Retrospective pilot study	45 patients	RRMS	ATZ vs. IFN or GA	N/A 60 months	Assess pRNFL and GCIPL changes	OCT	Significantly lower atrophy rate in ATZ‐treated patients		High
Lambe et al. (2021)	Observational study	250	RRMS	RTX vs. GA vs. NTZ vs. Healthy	2.8 year	Effects of rituximab on different retinal layers atrophy (pRNFL, GCIPL, INL, ONL)	OCT	Increased atrophy rates of the GCIPL during the first 12 months in RTX. After the 12‐month, RTX atrophy rates became similar to NTZ ‐treated patients and healthy control	Similar atrophy rate of RTX with GA in first 12 months and lower atrophy rate after 12 months	High
Miscioscia et al. (2022)	Blind, longitudinal Cohort study	75	36 PPMS vs. 39 Healthy	Ocrelizumab	Ocrelizumab 12 months	Assess retinal layer (pRNFL, GCIPL, INL, ONL, RPE, IRL) and brain changes	OCT MRI	Lower INL thinning rate in ocrelizumab‐treated patients		High
García‐Martín et al. (2010)	Longitudinal observational study	79 patients (155 eyes)	77RRMS 1PPMS 1SPMS	Without treatment vs. IFN‐1a vs. IFN‐1b vs. GA	12 months	Assess RNFL atrophy	Snellen chart Ishihara pseudoisochromatic visual field examination, optical coherence tomography (OCT), scanning laser polarimetry (Glaucoma Diagnosis) and visual evoked potentials	Similar atrophy rate between different drugs which was lower than untreated patients		Moderate
Sazonov et al. (2018)	Observational study	20	RRMS	—	GA 12 Months	Assess RNFL, AMT, GCIPL changes	OCT	GA reduced the rate of GCIPL and RNFL atrophy		High
Zivadinov et al. (2018)	Prospective, observational, single‐blinded, cohort study	100	60 RRMS patients vs. 40 healthy	GA‐treated RRMS vs. healthy	GA 24 months	Assess RFNL and TMV changes in GA‐treated RRMS	OCT	Similar retinal axonal degeneration in GA‐treated patients with healthy control		High
Ehrhardt et al. (2021)	Observational cohort study	230	RRMS	DMF vs. GA vs. NTZ	36 months	Assess GCIPL changes in RRMS	OCT	DMF has less thinning of the GCIPL than GA‐treated patients and greater than NTZ ‐treated patients		High
Wang et al. (2023)	A post hoc analysis of the FREEDOMS II study	885	RRMS	FTY	24 months	The association between baseline RNFL thickness and baseline clinical characteristics and clinical/MRI outcomes up to 24 months	OCT	Lower baseline RNFL thickness was associated with greater brain volume loss, higher total T2 lesion volume, and subsequent RNFL thinning up to 24 months.	Baseline RNFL thickness was not associated with annualized relapse rate and EDSS worsening	Moderate
Nolan et al. (2013)	Longitudinal observational study	60	RRMS	30 FTY‐RRMS vs. 30 no FTY‐RRMS	5 months (FTY) vs. 6 months (no FTY)	Assess macular volume MV and RNFLT thickness change	SD‐OCT	More increase of macular volume MV in the FTY group vs. the comparison group	No change in RNFLT thickness between either group	High
Fruschelli et al. (2019)	Longitudinal observational study	23	RRMS	FTY	12 months	Assess TMV and CFT change	SD‐OCT	No change in TMV at 3,6 or 12 months after treatment	No difference in TMV and CFT change among Patients with and without a history of ON	High
Garcia‐Martin et al. (2021)	Longitudinal Observational Cohort study	110	RRMS	78 FTY vs. 32 IFN	12 months	Asses visual function parameters (visual acuityVA, CSV, and color vision), average CMT, GCL thickness, and RNFL thickness	OCT, high‐contrast (2.5%) and low‐contrast (1.25%) visual acuity, contrast sensitivity vision (CSV) (using Pelli–Robson and CSV‐1000E tests), color vision (Farnsworth D‐15 and L'Anthony D‐15 desaturated tests)	Both groups showed GCL atrophy but the atrophy rate was higher in IFN at the 12‐month follow‐up than in FTY	RNFLT thickness paradoxically increased in the FTY group vs. reduction in the IFN group at the 12‐month follow‐up, the FTY group had increased average CMT and decreased visual acuityVA and CSV	High
Nørgaard et al. (2020)	Longitudinal observational study	190 eyes	RRMS	FTY	3–4 months	Asses TMV, TMT, CRT	OCT	Both TMV and TMT increased in the follow‐up	Change in TMT was clinically irrelevant and no correlation between change in CMT and TMV	High
D'Ambrosio et al. (2020)	Longitudinal observational study	60 RRMS patients	RRMS	FTY	24 months	Asses TMV, BCVA	OCT	Non‐significant increase in TMV		High
Karaküçük et al. (2020)	Longitudinal observational study	130	80 RRMS patients vs. 50 healthy	40 RRMS (FTY > 6 months) vs. 40 RRMS (FTY < 6 months) vs. 50 healthy (no groups had a hx of ON)	More than 6 months	Assess VDs, VDd, VDcc and CMT	Swept‐source OCTA	VDs and VDd values were higher in the FTY < 6 months group vs. other groups	No difference in VDcc between groups, CMT decreased in FTY > 6 months unlike. other groups	High

Abbreviations: AMT, Average macular thickness; ATZ, Alemtuzumab; BCVA, Best corrected visual acuity; CFT, central foveal thickness; CMT, central macular thickness; CMT, central macular thickness; CRT, Central retinal thickness; DMF, dimethyl fumarate; DMT, Disease Modifying Therapy; DMT, Disease Modifying Therapy; EDSS, Expanded Disability Status Scale; FTY, Fingolimod; GA, Glatiramer Acetate; GCIPL, ganglion cell–inner plexiform layer; GCL, ganglion cell layer; IFN, Interferon beta; INL, inner nuclear layer; IRL, inner retinal layer; NTZ, Natalizumab; OCT, optical coherence tomography; ON, Optic neuritis; ONL, Outer nuclear layer; PMS, progressive multiple sclerosis; PPMS, primary progressive multiple sclerosis; pRNFL, peripapillary retinal nerve fiber layer; RNFL, retinal nerve fiber layer; RPE, retinal pigment epithelium; RRMS, relapsing–remitting multiple sclerosis; RTX, Rituximab; SPMS, secondary progressive multiple sclerosis; TMT, Total macular thickness; TMT, Total macular thickness; TMV, Total macular volume; TMV, Total macular volume; VDcc, choriocapillaris vascular density; VDd, deep vascular density; VDs, superficial vascular density.

**TABLE 2:** Comparison of Retinal Layer Thickness by different DMTs.

Retinal layer	DMTs	Compared to healthy control	Compared to low and medium‐potency DMTs (IFN, GA, DMF)
GCIPL	Alemtuzumab	—	*p* < 0.0001 (63)
	Rituximab	0.006 (77)	*p* = 0.69 (77)
	Natalizumab	0.72 (61)	0.001 (62) < 0.001 (60) 0.001 (76)
	Fingolimod	0.73 (61)	< 0001 (61) < 0.001 (62)
pRNFL	Alemtuzumab	—	—
	Rituximab	—	—
	Natalizumab	—	0.004 (15)
	Fingolimod	0.014^a^ (61)	0.037^a^ (61)

Abbreviations: GCIPL, ganglion cell–inner plexiform layer; pRNFL, peripapillary retinal nerve fiber layer.

^a^ *p* < 0.01 was considered as significant.

### 3.1 Interferon Beta

Interferon beta, the first drug approved by the FDA for the treatment of MS in 1993 [52], offers various therapy options today, including subcutaneous and intramuscular formulations with different injection frequencies, ranging from every other day to every 2 weeks [52, 53].

Interferon beta is a naturally occurring polypeptide primarily produced by fibroblasts, and it operates through several mechanisms. These include the induction of antigen presentation downregulation, leading to the suppression of T cell activity; the induction of IL‐10, which directs the differentiation of CD4+ T cells towards a Th2 phenotype; and the reduction of T cell migration by decreasing adhesion molecules and matrix metalloproteinase 9 [54, 55]. Multiple studies have demonstrated that interferon beta effectively reduces the relapse rate in MS patients by approximately 30% [56, 57, 58, 59].

While interferon has been reported to be effective in reducing retinal atrophy [16], its impact on atrophy rate is relatively modest, and the rate of thinning is more pronounced compared to healthy individuals (*p* < 0.001 for subcutaneous IFN and *p* = 0.08 for intramuscular IFN) [60, 61]. Patients treated with interferon exhibit a higher atrophy rate compared to other DMTs, particularly in the case of retinal ganglion cells [60, 62]. Button et al. reported that patients receiving subcutaneous interferon displayed the highest rate of GCIPL atrophy (−0.54 μm/y) when compared to patients treated with other DMTs [60]. A study conducted by Chan et al. reported that, over a five‐year period, interferon‐treated patients had a higher rate of retinal nerve fiber layer (RNFL) (*p* = 0.0001) and GCIPL atrophy (*p* < 0.0001) compared to alemtuzumab‐treated patients [63]. Some studies have indicated that fingolimod provides better retinal protection than interferon (*p* < 0.001) [61, 64].

These outcomes may be attributed to the lower potency of interferon compared to other DMTs. Additionally, interferon therapy has been associated with side effects, including cotton wool spots, retinal hemorrhage, and retinopathy [65, 66].

### 3.2 Glatiramer Acetate

In 1997, the FDA granted approval for the use of Glatiramer acetate (GA) in the treatment of relapsing–remitting multiple sclerosis (RRMS). GA is a polymer composed of four amino acids, which mimic sequences found in myelin basic protein. These sequences bind to myelin‐specific autoantibodies, thereby reducing autoreactivity and promoting the development of a predominant Th2 phenotype in CD4+ T cells reactive to Glatiramer acetate. These T cells can accumulate in the central nervous system (CNS), ultimately altering the balance between proinflammatory and regulatory cytokines [67, 68, 69, 70, 71].

GA has demonstrated its efficacy in reducing the annual relapse rate by approximately 29% and the mean number of gadolinium‐enhancing lesions on MRI [72]. Furthermore, it has proven effective in decreasing the risk of new MRI lesions in clinically isolated syndrome (CIS) [73].

The effects of GA on retinal atrophy have been examined in several studies. Sazanov et al. reported that GA effectively reduced the rate of GCIPL atrophy after 12 months of treatment [74]. However, other studies have presented conflicting results. Zadinov et al. found no significant difference in RNFL and total macular atrophy between patients with RRMS treated with GA and healthy controls over a 24‐month period(*p* = 0.345 for optic‐neuritis–unaffected eye and *p* = 0.141 for optic‐neuritis–affected eye) [75]. Conversely, several studies have reported higher rates of retinal atrophy in GA‐treated patients compared to healthy controls [15, 60, 76]. Button and colleagues observed that while GA‐treated patients had a significantly lower atrophy rate than patients treated with subcutaneous interferon (IFN), they had a significantly higher rate than patients treated with natalizumab and healthy controls. Additionally, GA‐treated patients had a similar atrophy rate to patients treated with intramuscular IFN [60]. Other studies also reported that GA had a higher atrophy rate than natalizumab‐treated patients [15, 76, 77]. GA exhibited a similar atrophy rate to dimethyl fumarate (for GA −0.30 μm/y 95% confidence interval [CI] −0.38 to −0.22 μm/y vs. dimethyl fumarate −0.28 μm/y 95% CI −0.35 to −0.21 μm/y) [76].

Lambe et al. reported that the GCIPL atrophy rate in GA‐treated patients (−0.33 μm/y 95% CI −0.38 to −0.28 μm/y) was comparable to rituximab (−0.28 μm/y 95% CI ‐0.39 to −0.17 μm/y, *p* = 0.69) and lower than natalizumab (−0.17 μm/y 95% CI −0.27 to −0.07 μm/y). However, after 12 months, GA‐treated patients (−0.30 μm/y 95% CI −0.38 to −0.21 μm/y) had a greater atrophy rate compared to patients treated with natalizumab (−0.13 μm/y 95% CI −0.32 to +0.05 μm/y) and rituximab (−0.14 μm/y 95% CI −0.39 to +0.11 μm/y) [77]. Moreover, unlike rituximab, the atrophy rate in GA‐treated patients did not differ between the first and second year of treatment (−0.39 μm/y 95% CI −1.05 to +0.27 μm/y vs. −0.30 μm/y 95% CI −0.38 to −0.21 μm/y, *p* = 0.78) [77]. Chan et al. also reported that GA was less effective than alemtuzumab in reducing GCIPL thinning over a 5‐year period (*p* < 0.0001) [63].

## 4 Oral Medication

### 4.1 Sphingosine‐1‐Phosphate (S1P) Receptor Modulators

Fingolimod is the first orally administered drug approved in 2010 by the US Food and Drug Administration for the treatment of relapsing–remitting multiple sclerosis [1]. Its beneficial therapeutic effect in MS is due to the capacity of sequestrating lymphocytes into lymph nodes [2, 3] and preventing them from passing out of the lymphoid tissues into the peripheral circulation and the CNS. Thus, it stops lymphocytes from attacking myelin. Fingolimod is an effective drug, which significantly decreases the number of relapses in RR‐MS patients, slows disability progression, reduces inflammatory activity, and lowers the cerebral volume loss rate in MRI lesions [1, 4].

For the S1P1 receptor (highest affinity with fingolimod's phosphate ester metabolite), fingolimod initially operates as a functional agonist, which causes endocytic internalization [78], but in the long run acts as a functional antagonist because its sustained exposure causes a reduction in the S1P1 receptor numbers on the cell surface [79, 80]. In vitro studies have shown that this reduction persists even after discontinuation of treatment, suggesting a disturbed recycling of the receptor back to the plasma membrane [78, 81].

S1P receptors play more than one role, and besides regulation of the vascular endothelial barrier and intracellular junctions, they prevent degeneration of photoreceptors and ganglion cells [82, 83, 84]. Ceramide, the core lipid of sphingolipid metabolism, is a key factor in apoptotic cell death, and its increased levels are associated with the death of photoreceptors and retinal pigment epithelium cells [85]. Indeed, studies in animal models described that FTY inhibits ceramide synthase and blocks de novo ceramide production, suggesting its possible role in the protection of photoreceptors from apoptosis and delaying retinal degeneration [86, 87]. Therefore, the hypothesis that FTY might have a protective role in the retina should be considered [82].

Fingolimod is thought to affect the blood‐retina barrier in the retinal inner capillary network through S1P1 and S1P3 receptors [88]. In the retina, S1P receptor acts on endothelial cells to regulate barrier integrity, arterial tone, vascular permeability and tissue perfusion [89]. FTY treatment may induce the downregulation of adhesion complexes and subsequently increase retinal vascular permeability. This may result in fingolimod‐associated macular edema as well as other similar vascular retinopathies [90, 91, 92]. However, how fingolimod affects macular perfusion is unknown.

In a study by Nolan et al., it was reported that total macular volume (TMV) increased by a mean of 0.025 mm^3^ (95% CI +0.017 to +0.033, *p* < 0.001) in the fingolimod‐treated group over a mean follow‐up of 5 months compared to the insignificant change in the control group who received other therapies over a mean follow‐up of 6 months (−0.003 mm^3^ 95% CI −0.009 to +0.004, *p* = 0.47). Additionally, 74% of eyes in the fingolimod group exhibited an increase in TMV vs. the 37% of eyes in the comparison group, and more than half of the fingolimod patients had commenced therapy because of a flare‐up or progressive disease, which makes it possible that disease activity, not the fingolimod itself, led to an increase in TMV. It should be noted that there was no significant change in visual acuity during the observation period. Moreover, although there was a greater reduction in RNFL thickness in the control group, this difference did not reach statistical significance (−0.03 μm 95% CI −0.58 to 0.52 μm vs. −0.82 95% CI −1.43 to −0.60 μm, *p* = 0.11) [93]. This increase in TMV may be attributed to a known side effect of fingolimod, which elevates the risk of developing microcystic macular edema [94]. Furthermore, two studies noted that patients treated with fingolimod exhibited lower retinal thickness [95, 96].

Nørgaard et al. reported that 3–4 months after the initiation of fingolimod showed that both TMV and total macular thickness (TMT) increased during 3–4 months follow‐up (+0.05 mm^3^ for TMV and +19.7 μm for TMT, *p* < 0.001) [97]. Similar findings were reported by d'Ambrosio and colleagues during 3–4 months, although this increase was not significant (+ 0.01 mm^3^, *p* = 0.67) [98]. However, after 2 years of follow‐up, there was a non‐significant decrease in TMV (− 0.08 mm^3^, *p* = 0.07) [98].

Another recent study published by Fruschelli et al. on 23 MS patients longitudinally recorded with spectral domain OCT found no statistically significant change in TMV at 3, 6, or 12 months after initiation of fingolimod independent of previous ON history (*p* = 0.96). There was no significant change in best‐corrected visual acuity, and comparing patients with and without a history of ON, no statistically significant change was found for TMV (*p* = 0.96) and central foveal thickness (*p* = 0.99) [99].

In a study by Wang et al., it was found that, compared to a placebo, fingolimod 0.5 mg effectively reduced the rate of inferior quadrant of pRNFL thickness atrophy in patients with RRMS after 24 months (*p* = 0.047) [100]. Furthermore, in a 12‐ and 24‐month follow‐up period, fingolimod proved to be more effective than interferon [61, 62, 64]. Additionally, patients treated with fingolimod did not exhibit a significantly different rate of GCIPL atrophy compared to a healthy population (for healthy control −0.25 μm 95% CI −1.3 to 1 vs. −0.22 μm 95% CI −0.21 to 0.09, *p* = 0.73) [61]. You et al. reported that patients treated with fingolimod had similar rates of atrophy as those treated with Glatiramer acetate and natalizumab in various OCT measurements, except for GCIPL atrophy, where GA‐treated patients had a higher atrophy rate [62].

### 4.2 Teriflunomide

Teriflunomide received FDA approval in 2012 for the treatment of RRMS and active secondary progressive multiple sclerosis (SPMS). Teriflunomide is the active metabolite of leflunomide [4]. It functions as an immunomodulatory agent that selectively and reversibly inhibits the mitochondrial enzyme dihydroorotate dehydrogenase, thus disrupting de novo pyrimidine synthesis. This disruption results in the reduced proliferation of dividing cells that rely on de novo pyrimidine synthesis for their expansion [101]. The therapeutic effect is achieved through a reduction in the number of circulating lymphocytes and the inhibition of the proliferation of activated lymphocytes [101, 102].

Teriflunomide has been demonstrated to reduce relapse rates and disability progression by approximately one‐third. It also significantly decreases gadolinium‐enhancing lesions in MRI scans by about 80% [103, 104].

To date, there have been no studies examining the effect of teriflunomide on retinal layers in human MS patients. However, Groh and colleagues conducted an animal study in which a dosage similar to that used in human MS patients was administered. The study found that teriflunomide effectively attenuated CD8+ cytotoxic T cells and promoted the proliferation of CD8+ CD122+ PD‐1+ regulatory T cells. These effects resulted in a reduction of neuronal damage in the retinotectal system in MS mice. Additionally, teriflunomide slowed the thinning of the innermost retinal composite layer, which includes the nerve fiber layer, ganglion cell layer, and inner plexiform layer. This slowing of retinal thinning was correlated with improved clinical outcomes, suggesting a neuroprotective effect [105].

### 4.3 Fumarates

Dimethyl fumarate (DMF) is one of the most well‐known drugs in this category and received FDA approval for MS treatment in 2013 [53, 106]. DMF activates the nuclear factor (erythroid‐derived 2)‐like 2 (Nrf2) pathway [107]. This activation leads to an anti‐inflammatory effect by shifting cytokine production away from interferon gamma and TNFα, favoring the production of IL4 and IL5 [108]. Additionally, DMF upregulates Nrf2‐dependent antioxidant genes in patients with MS [107]. It is often used as a first‐line treatment for MS [52] and has been effective in reducing relapse rates, disability progression, and MRI gadolinium‐enhancing lesions. However, there have been no significant differences observed in the time to disability progression [109].

In a recent study comparing the effects of DMF with glatiramer acetate and natalizumab, it was found that patients treated with DMF experienced less thinning of the GCIPL compared to GA‐treated patients 12 months after DMT initiation (−0.33 μm/y 95% CI −0.42 to −0.24 μm/y vs. −0.25 μm/y 95% CI −0.36 to −0.14), which was not significant. However, GCIPL thinning was greater in comparison to healthy controls (−0.28 μm/y 95% CI −0.35 to −0.21 vs. −0.15 μm/y 95% CI −0.20 to −0.10, *p* = 0.008) [76].

You et al. considered DMF as a high‐potency DMT and reported lower rates of RNFL and GCIPL thinning in high‐potency DMTs compared to lower‐potency drugs [62].

## 5 Monoclonal Antibodies

### 5.1 Alemtuzumab

Alemtuzumab received FDA approval for the treatment of MS in 2014. This humanized monoclonal antibody selectively targets the CD52 antigen, which is prominently present on the cell surface of T and B lymphocytes [53, 110]. CD52 is also expressed on natural killer (NK) cells, monocytes, and certain other granulocytes [111, 112]. When alemtuzumab binds to CD52, it initiates cellular lysis through mechanisms involving antibody‐dependent cytolysis and complement‐mediated processes. This leads to lymphocyte depletion and subsequent repopulation [110]. Alemtuzumab has proven to be effective in reducing relapses, slowing disease progression in patients with RRMS, and decreasing white matter lesions detected by MRI [113, 114].

While one study specifically investigated alemtuzumab's impact on changes in retinal layers, other studies examined it in combination with other drugs. Chan et al. conducted a retrospective cohort study to compare alemtuzumab with first‐line injectable treatments (interferon‐beta or glatiramer acetate). Despite alemtuzumab‐treated patients having more disability and disease activity at baseline, the study revealed that RNFL thinning (−0.88 μm 95% CI −2.63 μm to +0.86 μm for the alemtuzumab treated group and −3.65 μm 95% CI −5.40 to −1.89 μm for interferon‐treated and GA‐treated patients, *p* = 0.0001) and GCIPL volume (+0.013 mm^3^ 95% CI −0.006 to +0.032 mm^3^ for the alemtuzumab treated group and −0.052 mm^3^ 95% CI −0.070 to −0.034 mm^3^ for interferon‐treated and GA‐treated patients, *p* < 0.0001) loss were significantly lower in alemtuzumab‐treated patients compared to the first‐line treated group over a period of 60 months. However, changes in RNFL thickness were significantly higher in eyes with a history of ON, suggesting that the treatment has less of an effect on RNFL neurodegeneration after an episode of ON compared to eyes without a history of ON. These findings indicate a potential neuroprotective effect of alemtuzumab [63]. It was also reported that alemtuzumab has a similar protective effect to mesenchymal stem cell transplantation [115].

Similarly, you and colleagues reported lower rates of RNFL and GCIPL thinning in high‐potency DMTs compared to lower‐potency drugs [62].

### 5.2 Rituximab

Rituximab is a monoclonal antibody that combines mouse and human components and is specifically designed to target CD20, a cell surface marker found on pre‐B and B cells. This targeting leads to the depletion of pre‐B cells and mature B cells [111].

Although not approved by the FDA, rituximab has been used in the treatment of multiple sclerosis for many years. It has proven effective in patients with RRMS and primary progressive multiple sclerosis (PPMS) [116].

Rituximab has been found to significantly reduce the relapse rate and decrease the occurrence of MRI gadolinium‐enhancing lesions, with a reduction of up to 91% [117, 118].

In an observational study conducted by Lambe and colleagues, patients with RRMS who were treated with a single disease‐modifying therapy and healthy controls were monitored with serial OCT. The study revealed that rituximab initially increased the atrophy rates of the GCIPL during the first 12 months of treatment (−0.69 μm/y 95% CI −1.09 to +0.29 μm/y). However, after the 12‐month mark, rituximab's atrophy rates (−0.14 μm/y 95% CI −0.39 to +0.11 μm/y)became similar to those of patients treated with natalizumab (−0.13 μm/y 95% CI −0.32 to +0.05 μm/y) and healthy controls (−0.15 μm/y 95% CI −0.18 to −0.12 μm/y). Possible explanations for this phenomenon include the additive neuroprotective effect of rituximab, especially as the therapeutic effect occurring between months 7 and 12 exceeded that of the first 6 months. Another explanation could involve a late‐onset neuroprotective effect of rituximab or pseudoatrophy due to reduced inflammation and subsequent edema after the initiation of therapy [77]. As rituximab is considered a high‐potency disease‐modifying therapy, patients treated with rituximab experienced lower rates of thinning in the RNFL and GCIPL compared to lower‐potency drugs [62].

### 5.3 Ocrelizumab

In 2017, Ocrelizumab received FDA approval for the treatment of RRMS. Notably, it is the only drug approved by the FDA for the treatment of PPMS as well [119, 120]. Ocrelizumab is a humanized monoclonal antibody that specifically targets CD20 B cells. It induces B cell depletion through processes of antibody‐dependent cytolysis and complement‐mediated lysis [120]. Ocrelizumab selectively removes B cells expressing CD20 while preserving pre‐existing humoral immunity and the capacity for B‐cell reconstitution. This B‐cell depletion results in a significant interruption in B‐cell trafficking from the periphery to the CNS, reduced B cell antigen presentation to T cells, modulation of proinflammatory cytokine secretion by B cells, and reduced activation and differentiation into immunoglobulin‐secreting plasmablasts [121, 122]. As a result, B cell depletion is almost complete by the end of the second week [120]. This therapy also leads to reductions in the concentration of neurofilament light chain, as well as in the numbers of CD19+ B cells and CD3+ T cells [116].

Ocrelizumab has demonstrated its high effectiveness in reducing disability and relapse rates and has a significant impact on the attenuation of MRI gadolinium‐enhancing lesions in RRMS [119, 120, 123, 124].

In a recent study, it was shown that ocrelizumab significantly slowed the rate of INL thinning in PPMS. This study hypothesized that ocrelizumab induces the suppression of brain inflammation, which may also protect neuronal cells in the retina from neurodegeneration. This neurodegeneration can occur due to local inflammation and trans‐synaptic degeneration along the visual pathway [35]. Similarly, you and colleagues reported lower rates of thinning in the RNFL and GCIPL in high‐potency DMTs compared to lower‐potency drugs [62].

### 5.4 Natalizumab

Natalizumab, an FDA‐approved medication since 2004, is a humanized monoclonal antibody that alters immune cell migration by blocking α‐4 β‐1 and β‐7 integrins. This mechanism inhibits the infiltration of leukocytes across the endothelium into the brain [53].

The α‐4 β‐1 integrin, expressed on lymphocytes and involved in transmigration across endothelial cells into the CNS, plays a key role in the drug's action [125].

Natalizumab has been shown to reduce relapse rates by 68% and new gadolinium‐enhancing lesions by 83%, with a 42% interruption of disability progression over a two‐year period [126, 127].

Many studies have considered Natalizumab to be the most effective DMT for retinal layer atrophy [15]. It was reported that Natalizumab significantly reduced pRNFL thinning compared to first‐line drugs (*p* = 0.004) [15]. In another study, patients treated with Natalizumab had lower GCIPL thinning (−0.45 μm/y 95% CI −0.78 to −0.13 μm/y) compared to those treated with rituximab (−0.69 μm/y 95% CI −1.09 to +0.29 μm/y) during the first 12 months, which was not significant (*p* = 0.13). However, after 12 months, the atrophy rate (−0.13 μm/y 95% CI −0.32 to +0.05 μm/y) was comparable to rituximab‐treated patients (−0.14 μm/y 95% CI −0.39 to +0.11 μm/y) but was lower than GA‐treated patients (−0.30 μm/y 95% CI −0.38 to −0.21 μm/y) [77]. Button et al. conducted a cohort study and demonstrated that Natalizumab reduced the GCIPL atrophy rate more than other DMTs, with a GCIPL thinning rate of −0.17 mm/year in Natalizumab‐treated patients over approximately 3 years, which was the lowest compared to patients receiving GA and IFN‐b‐1a (*p* = 0.035 for GA and *p* = 0.001 for subcutaneous IFN) [60]. Ehrhardt et al. also reported that Natalizumab‐treated patients with RRMS were comparable to that of healthy individuals (0.28 μm/y 95% CI −0.35 to −0.21 μm/y vs. −0.15 μm/y 95% CI −0.20 to −0.10 μm/y) and 12 months after treatment initiation had a lower GCIPL atrophy rate (−0.14 ± 0.08 μm/y) compared to DMF (−0.25 μm/y 95% CI −0.36 to −0.14 μm/y) and GA (−0.33 μm/y 95% CI −0.42 to −0.24 μm/y), which was significant for GA (*p* = 0.001) [76]. However, not all studies have found such positive results, as Sotirchos et al. conducted a large cohort study and reported that Natalizumab did not slow the thinning of the INL and ONL in RRMS patients [25]. Additionally, in another study, there was no significant difference between Glatiramer acetate, Fingolimod, and Natalizumab in terms of retinal layer atrophy [62].

It is worth noting that greater brain atrophy occurs during the first year of Natalizumab treatment, but this phenomenon lessens in subsequent years. This has been linked to the resolution of pseudoatrophy [128, 129]. It is important for prospective studies to determine whether a similar phenomenon may occur in the retina and to understand the dynamics of retinal layer atrophy following treatment initiation.

## 6 Conclusion

In conclusion, all DMTs have been shown to reduce the rate of retinal layer atrophy, but their effects vary. Natalizumab has the highest effect, and the atrophy rate is similar to that of healthy people, followed by other high‐potency DMTs such as rituximab, alemtuzumab, fingolimod, and dimethyl fumarate, which were more effective than low‐potency DMTs like glatiramer acetate and interferons. Interferon‐treated patients had the highest atrophy rate, which was greater in subcutaneous forms. Only one study included progressive multiple sclerosis, and ocrelizumab was shown to be effective in reducing the atrophy rate.

The mechanisms by which disease‐modifying therapies affect retinal thickness are not yet fully understood, as each DMT appears to protect the retina through distinct mechanisms.

DMF is thought to promote the survival of retinal ganglion cells (RGCs) by increasing the density of tubulin β3‐positive RGCs and enhancing immunoreactivity for Nrf2 and heme oxygenase‐1 (HO‐1), a potent antioxidant enzyme. These findings suggest that DMF supports RGC survival after optic neuritis, possibly via the Nrf2/HO‐1 pathway [130, 131]. Fingolimod exerts both anti‐inflammatory and neuroprotective effects, preventing further damage to small RNFL axons [100]. In the retina, the S1P receptor regulates blood‐retinal barrier integrity, vascular permeability, and tissue perfusion by acting on endothelial cells. Fingolimod downregulates adhesion complexes, increases vascular permeability, and can lead to macular edema and retinopathy [89, 91, 92]. Additionally, it preserves neuronal viability and retinal function by promoting the proliferation and differentiation of retinal progenitor cells into photoreceptors, enhancing their survival through oxidative stress mechanisms [82, 83, 84]. Fingolimod also inhibits ceramide synthase and blocks de novo ceramide production, preventing ceramide accumulation in the retina, protecting photoreceptors from apoptosis, and delaying retinal degeneration [86, 87]. Collectively, fingolimod protects the retina by preventing photoreceptor and ganglion cell degeneration and maintaining blood‐retinal barrier integrity [132].

Alemtuzumab has been shown to slow neurodegeneration in retinal structures [133] and may reduce retinal layer atrophy by promoting remyelination [133, 134]. Rituximab inhibits TRPM2 in human retinal epithelial cells, which reduces inflammatory cytokine levels and apoptosis [135]. Natalizumab partially reduces microglia in the GCIPL [136]. Since microglia are a key source of proinflammatory cytokines and play a significant role in demyelination and the smoldering state of the disease [137, 138], this effect may contribute to its therapeutic benefits. Furthermore, natalizumab slows neurodegeneration and neuroaxonal damage [15, 77].

The number of studies that have investigated the effects of different drugs on retinal layer atrophy is limited. These studies have primarily measured longitudinal changes rather than absolute volume, which is more beneficial [139]. Additionally, the majority of these studies have focused on different layers of the retina. These studies have mainly investigated first‐line therapies and the relapsing–remitting form of the disease. However, there are several limitations in the existing literature on the effects of different drugs on retinal thickness and its layers. Most of these studies have small sample sizes, and because retinal layer atrophy is influenced by multiple factors, subgroup analyses are not feasible. Additionally, controlling for confounding variables such as smoking, BMI, disease severity, disease duration, age, treatment duration, and other comorbidities is often not possible.

Moreover, more studies are needed to assess the effects of different DMTs in progressive multiple sclerosis (PMS), as only one study has specifically focused on the progressive form of the disease. The nature of PMS differs from RRMS, and the findings regarding retinal layer changes have been inconsistent [46, 140]. Another limitation is the variation in the retinal layers measured across different studies. It is necessary to identify the most relevant layer correlated with disease progression and disability and focus future research on that layer. Additionally, we recommend using normal values of layer thickness rather than raw measurements in future studies.

Given the current limitations in data and the recent recommendations for early treatment in radiologically isolated syndrome (RIS) and CIS, comparative studies, particularly those considering retinal thickness, could provide more robust evidence. Furthermore, studies on newly approved drugs are limited. We recommend investigating and assessing new DMTs such as Siponimod, Ozanimod, and Cladribine in long‐term, longitudinal studies.

For a better understanding of the effects of different DMTs on the retina in MS, more longitudinal studies with longer follow‐up and larger populations, while controlling for other confounding factors, are warranted.

## Conflicts of Interest

The authors declare no conflicts of interest.

## Transparency Statement

The corresponding author affirms that this manuscript is an honest, accurate, and transparent account of the study being reported; that no important aspects of the study have been omitted; and that any discrepancies from the study as planned (and, if relevant, registered) have been explained.

## Data Availability

The data that support the findings of this study are available from the corresponding author upon reasonable request.
